# Trends in Physics Contributions to the 'Red Journal': A 30-year Journey and Comparison to Global Trends

**DOI:** 10.7759/cureus.3012

**Published:** 2018-07-20

**Authors:** Ravindra Yaparpalvi, Nitin Ohri, Wolfgang A Tomé, Shalom Kalnicki

**Affiliations:** 1 Radiation Oncology, Montefiore Medical Center/Albert Einstein College of Medicine, Bronx, USA

**Keywords:** radiation therapy, time-trend, igrt, medical physics, intensity-modulated radiotherapy (imrt), evolution, technology, brachytherapy, sbrt

## Abstract

Introduction: In this study, we catalogued physics contributions in the Red Journal over the past three decades and compared publication trends with global publication trends.

Methods: We used the website of the Red Journal (International Journal of Radiation Oncology, Biology, and Physics) to access physics contributions published between 1988 and 2017. The contributions were catalogued following taxonomy guidelines endorsed by the American Association of Physicists in Medicine. From each issue, publications classified as “Physics Contributions” or as “Technical Innovations” or listed a physicist as one of the primary authors was indexed. Results are presented using descriptive statistics; chi-square $\chi$^2^ testing were utilized to examine trends in contributions over 10-year time intervals. For global trend comparison of Red Journal physics contributions, we utilized PubMed database to obtain publication counts on the topics of interest.

Results: A total of 2,852 physics contributions were indexed (86 volumes and 436 issues). Overall, 76% of contributions were photon-beam therapy applications, 15% brachytherapy, 7% particle-beam therapy, and 3% electron-beam therapy. $\chi$^2^ analyses revealed significant changes in this distribution over time (p<0.001). Brachytherapy accounted for 23% of publications in the first decade, compared to 7% in the third decade. Particle beam therapy accounted for 4% of publications in the first decade and 12% in the third decade. Among treatment techniques, three-dimensional conformal radiation therapy (3D-CRT) accounted for 64% of contributions in the first decade, compared to 3% in the third decade. Intensity-modulated radiation therapy (IMRT)/volumetric modulated arc therapy (VMAT) accounted for 4% in the first decade, compared to 54% in the third decade. Significant increases in the proportions of studies focused on motion management, functional imaging for treatment planning, and radiation safety/quality assurance during the third decade were observed (p<0.001).

Conclusion: Trends of physics publications in the Red Journal and globally, in general, largely mirror technological advances in the field of radiation oncology. These changes reflect a technological transition in the field over three decades from beam's-eye–view designed static treatment ports to functional imaging and knowledge-based treatment planning with biological dose optimization and real-time tumor tracking.

## Introduction

The International Journal of Radiation Oncology, Biology, and Physics (IJORBP), commonly referred to as ‘The Red Journal’ in the radiation oncology field, is the official journal of the American Society for Radiation Oncology (ASTRO) and publishes investigations related to radiation oncology, radiation biology, and medical physics. This journal has a 2017 impact factor of 5.133 and in addition to radiation therapy clinical practice and advances, it publishes advances related to radiation oncology physics in several areas such as radiation dosimetry, normal tissue protection and modeling, brachytherapy, particle beam therapy, and imaging etc. In this study, we sought to catalog physics contributions in this journal and characterize the evolution of physics contributions over the past three decades.

## Materials and methods

We utilized the ScienceDirect database ( https://www.sciencedirect.com) (Elsevier, Amsterdam, The Netherlands), the publisher of the Red Journal to access physics research contributions from 1988 through 2017 (http://www.redjournal.org/). The contributions were catalogued as per taxonomy guidelines, recently adopted by the Medical Physics Journal (official journal of the American Association of Physicists in Medicine) (https://medphys.msubmit.net/html/taxonomy.pdf). Contents of each Red Journal issue, beginning at Volume 14, Issue 1 (1988) ending at Volume 99, Issue 5 (2017) were manually reviewed for indexing physics contributions. From each issue, publications classified by the Red Journal as “Physics Contributions”, “Technical Innovations”, and any contributions with a physicist as a primary author (based on academic degree and author affiliation within the institution), were manually catalogued. Editorials, letters to the editor, review articles, abstracts and ASTRO conference proceedings were excluded from the data collection.

Data were divided into decade intervals Decade One (1988 -1997), Decade Two (1998-2007) and Decade Three (2008-2017) respectively. Descriptive statistics were used for data analyses and the chi-square ($\chi$2) testing was utilized to compare trends in publication counts over ten-year periods. P-values <0.05 were considered statistically significant.

For global trend comparison of physics contributions on the topics of our study interest from taxonomy classification, we utilized PubMed database (https://www.ncbi.nlm.nih.gov/pubmed/). We collected publication counts for the study years (1988-2017) by performing PubMed search using relevant search terms per study interest. The CSV (comma space value) files in each case were then directly imported for data analyses and for comparison with the Red Journal publication counts.

## Results

**Figure 1:**
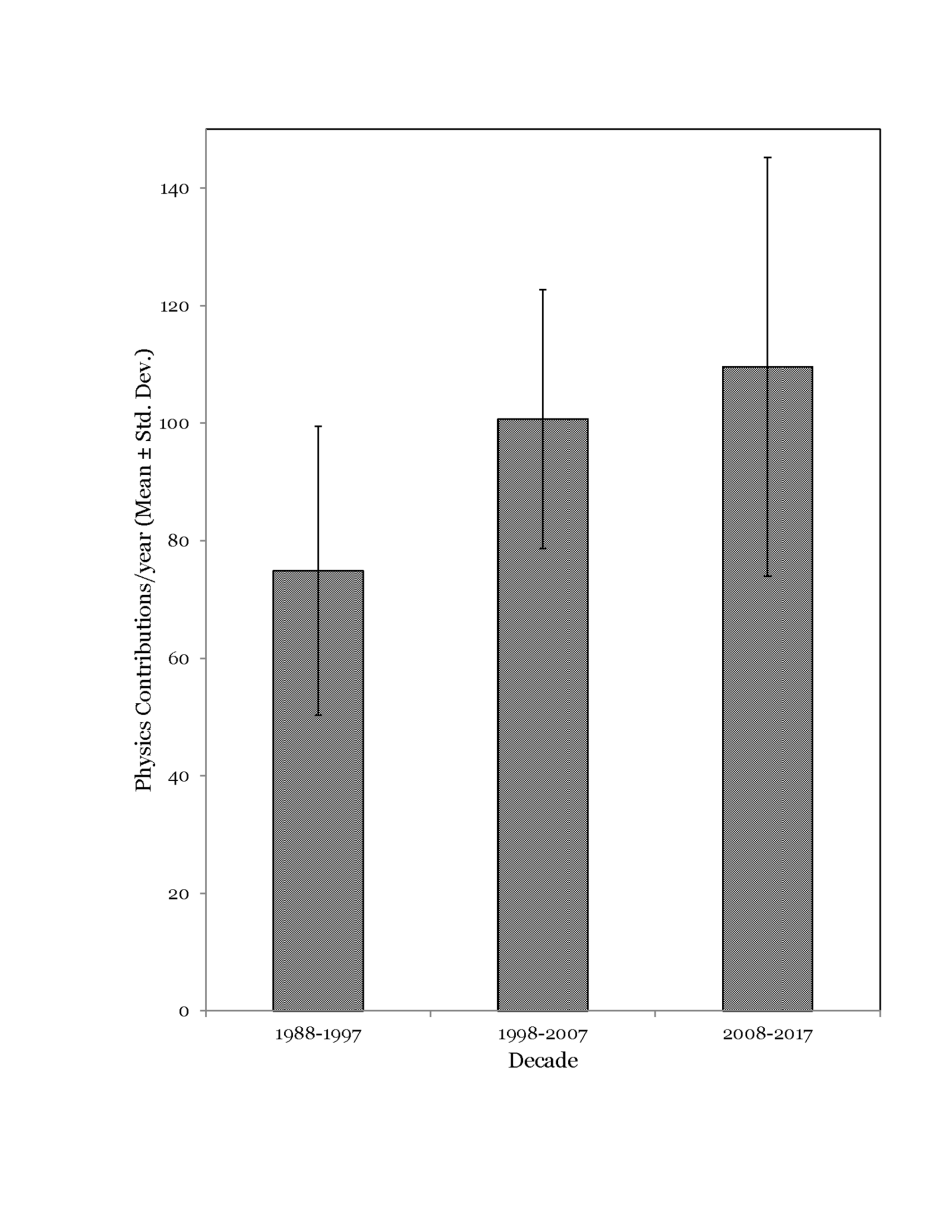
Average number of physics contributions/year for the three decades studied

A total of 2,852 physics contributions were indexed from 86 volumes comprising of 436 issues over the 30-year time period (1988-1997, N= 749; 1998-2007, N= 1007; 2008-2017, N=1111). The number of physics contributions increased by 34.5% in the second decade compared to the first decade and by 10.3% in the third decade in comparison to the second. The average number of physics contributions/year for the three decades studied is presented in Figure 1. The average number of physics contributions per issue was median 6 (inter-quartile range (IQR) = 5.3–7.9). The number of physics contributions per issue increased every decade: 1988-1997 (median 5, IQR = 4.0–7.1); 1998-2007 (median 6, IQR= 5.6–8.0); and 2008-2017 (median 7, IQR= 5.6–8.1) respectively.

Evaluating total physics contributions, for the 30-year period, 75% of the contributions were related to applications concerning photon beam treatment modality, 13% were related to brachytherapy, 7% addressed particle beam therapy, and 3% of the topics were on electron beam therapy. Remainder (2%) of the contributions was distributed among non-ionizing (hyperthermia and ultrasound) and radiopharmaceutical therapies.

**Figure 2:**
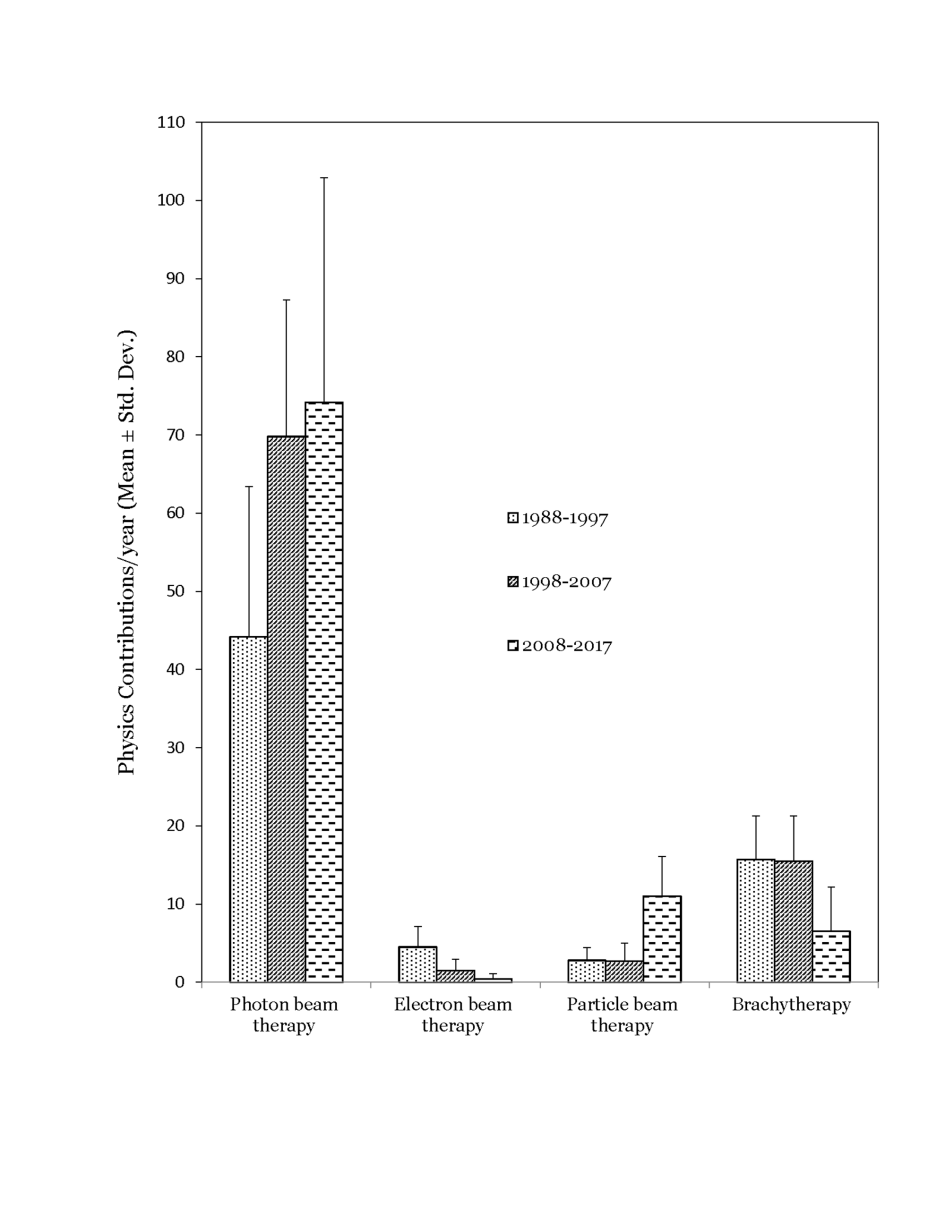
Changes in the number of physics contributions related to treatment modality over the three decades studied

$\chi$^2^ analyses revealed significant changes in the physics contributions topic distribution over time (p<0.001). Brachytherapy accounted for 23% of publications in the first decade, compared to 7% in the third decade. Electron beam radiotherapy accounted for 7% of publications in the first decade and 0.4% in the third decade. Particle beam therapy accounted for 4% of publications in the first decade and increased to 12% in the third decade (Figure 2).

**Figure 3:**
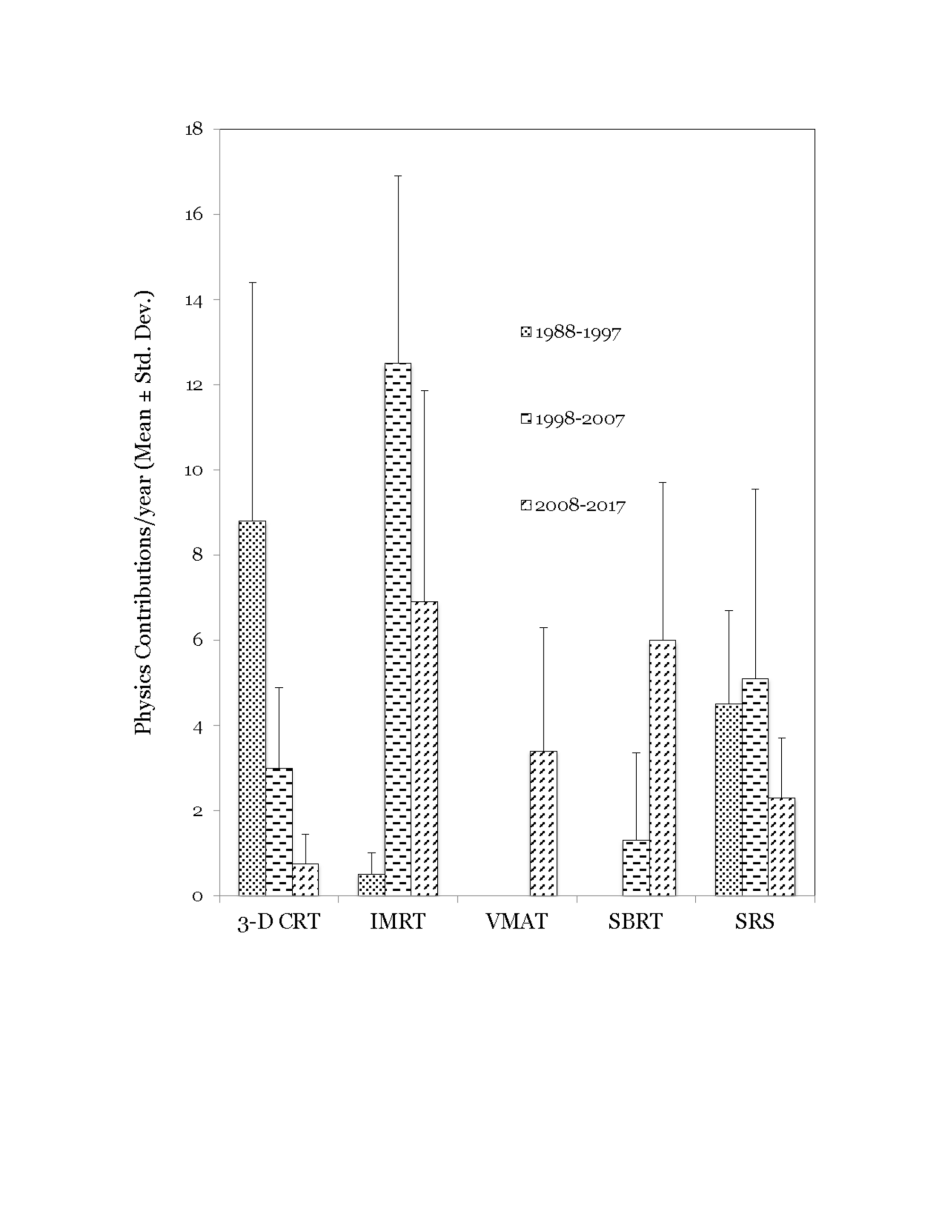
Changes in the number of physics contributions related to treatment technique over the three decades studied

Among physics contributions on treatment techniques, three-dimensional conformal radiation therapy (3D-CRT), intensity-modulated radiation therapy (IMRT), volumetric modulated arc therapy (VMAT), stereotactic radiosurgery (SRS) and stereotactic body radiation therapy (SBRT), there were significant changes over time (p<0.001). 3D-CRT related contributions accounted for 64% of these publications in the first decade, compared to 3% in the third decade. IMRT/VMAT topics accounted for 4% of publications in the first decade, compared to 54% in the third decade. SRS related contributions decreased 3-fold while SBRT related contributions increased 5-fold in the third decade compared to second decade (Figure 3).

There was a shift in the distribution of other topics, with significant increases during the second and third study decades, the proportion of physics contributions focused on imaging (computed tomography (CT), positron emission tomography (PET), and magnetic resonance imaging (MRI)), outcome/toxicity modeling (normal tissue complication probability (NTCP), tissue complication probability (TCP)), target motion and management, functional imaging for treatment planning, and radiation safety/quality assurance (including audits and failure mode and effects analysis (FMEA ))(Table 1).

**Table 1:** Changes in the number of physics contributions focused on imaging, target motion, modeling and safety CT: computed tomography; PET: positron emission tomography; MRI: magnetic resonance imaging; NTCP: normal tissue complication probability; TCP: tissue complication probability; FMEA: failure mode and effects analysis; QA: quality assurance.

DECADE	1988-1997	1998-2007	2008-2017	p-value
Imaging (CT, PET, MRI)	21	55	62	0.016
Motion and Management	8	120	107	<0.00001
Modeling - NTCP, TCP	20	89	90	<0.00001
Functional Imaging -Treatment Planning	2	32	95	<0.00001
Radiation Safety, Audits, FMEA, QA	3	15	60	<0.00001

**Figure 4:**
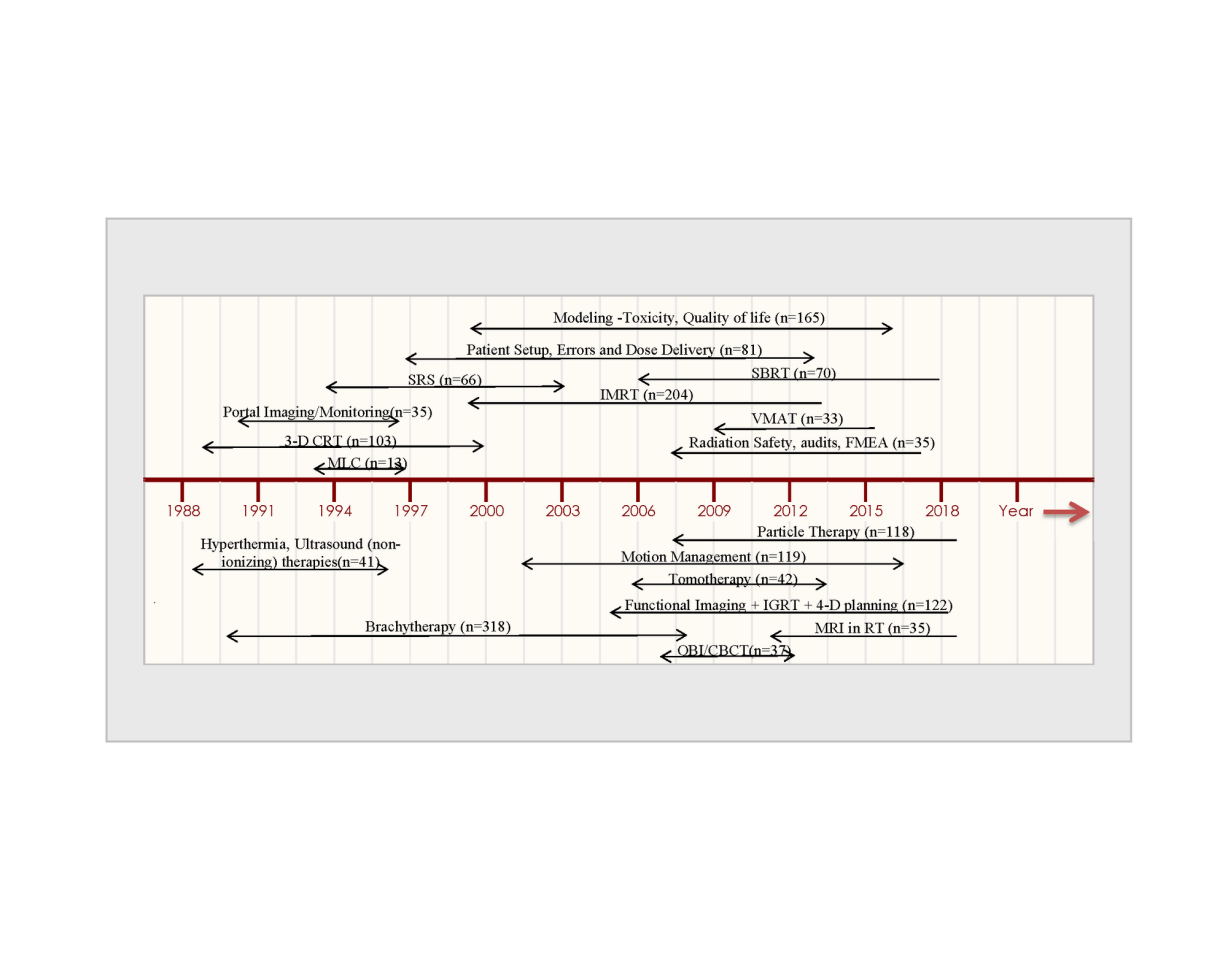
Time-trend depiction of increases in physics contribution counts on various topics that occurred at different time intervals of our study period SRS: stereotactic radiosurgery; SBRT: stereotactic body radiation therapy; IMRT: intensity-modulated radiation therapy; 3D-CRT: three-dimensional conformal radiation therapy; VMAT: volumetric modulated arc therapy; MLC: multi-leaf collimation; IGRT: image-guided radiation therapy; MRI: magnetic resonance imaging; RT: radiation therapy; OBI: on-board imaging; CBCT: cone-beam computed tomography.

Increases in physics contribution counts on various topics that occurred at different time intervals of our study period are summarized in a time-line trend fashion in Figure 4. 

**Figure 5:**
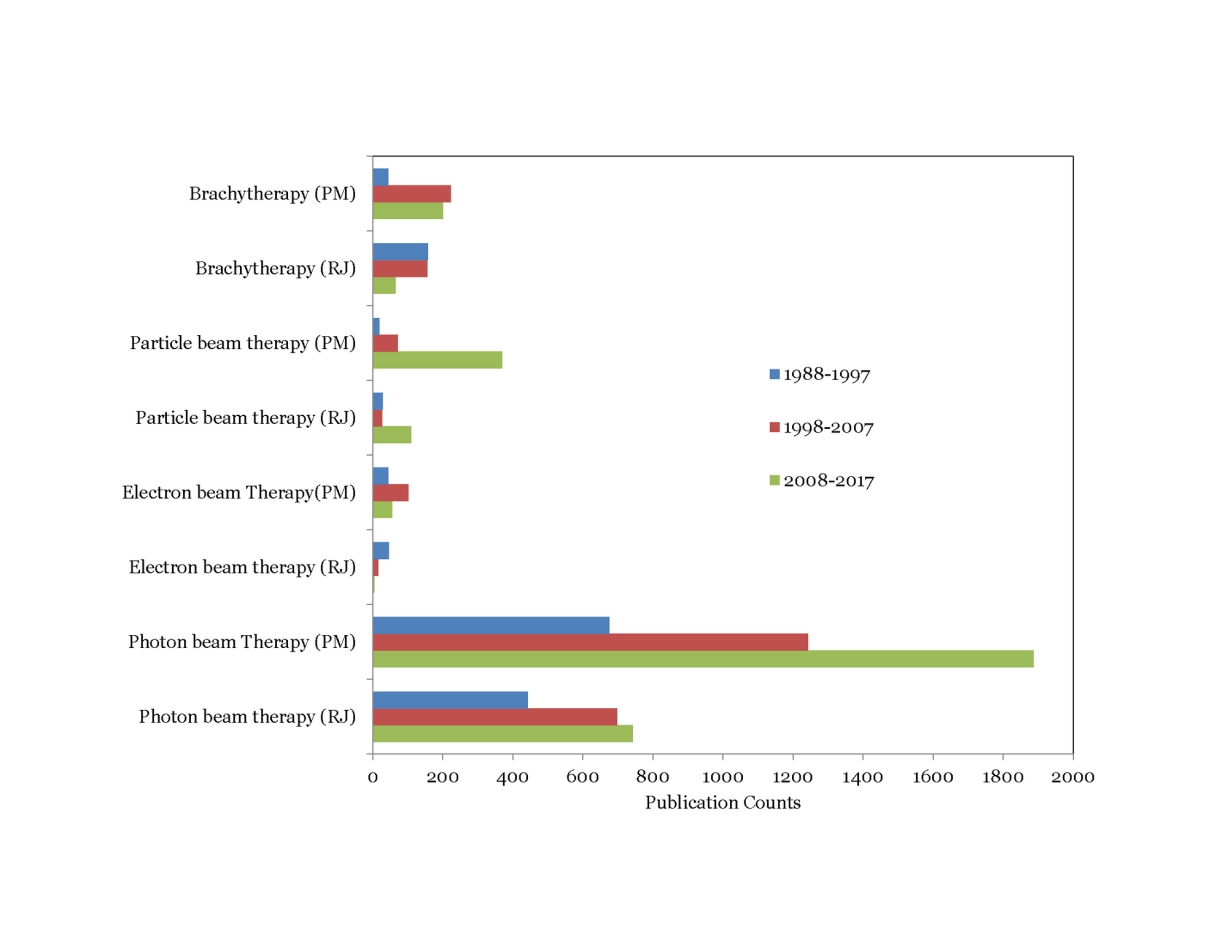
Comparison of publication trends related to the treatment modality between Red Journal (RJ) and PubMed (PM)

**Figure 6:**
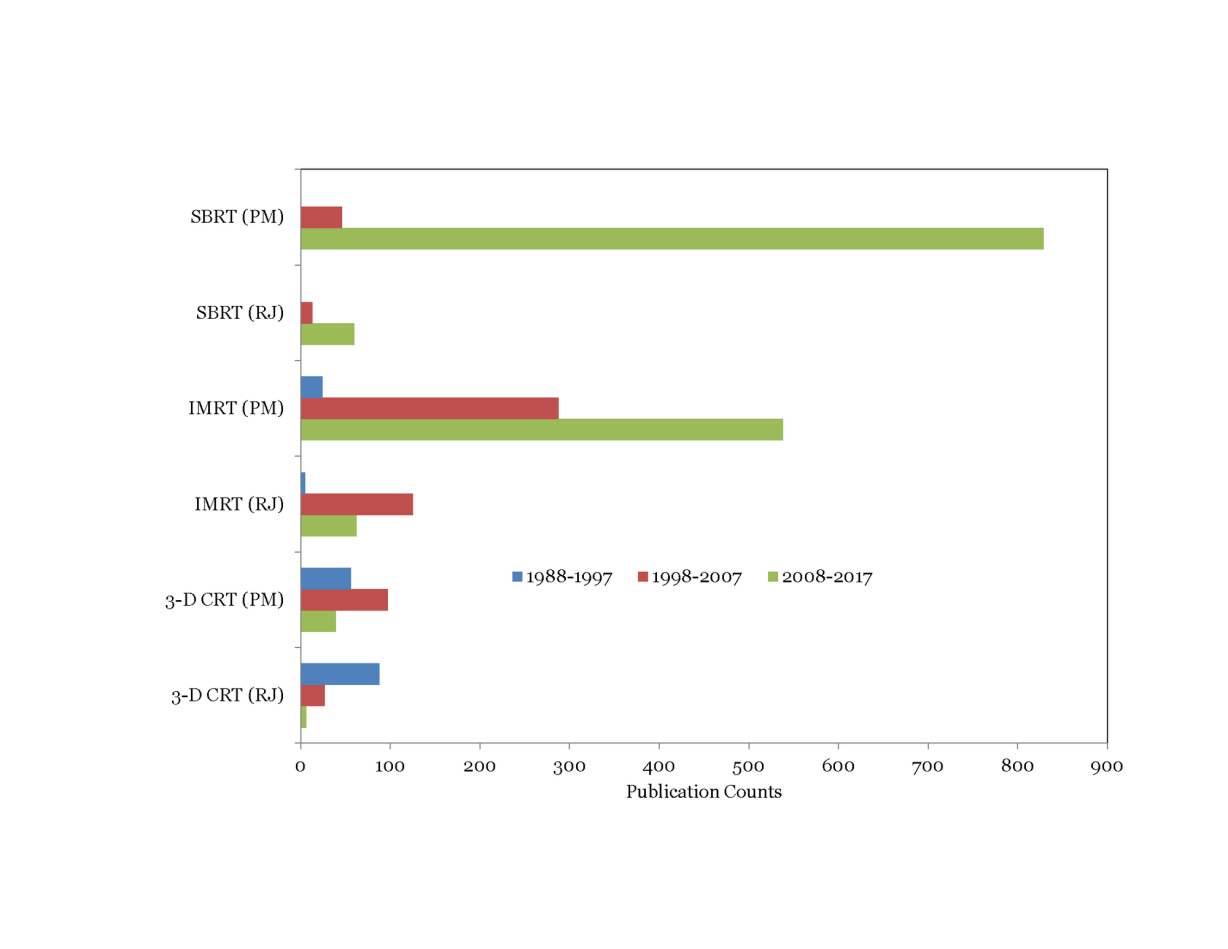
Comparison of publication trends related to the treatment technique between Red Journal (RJ) and PubMed (PM)

**Figure 7:**
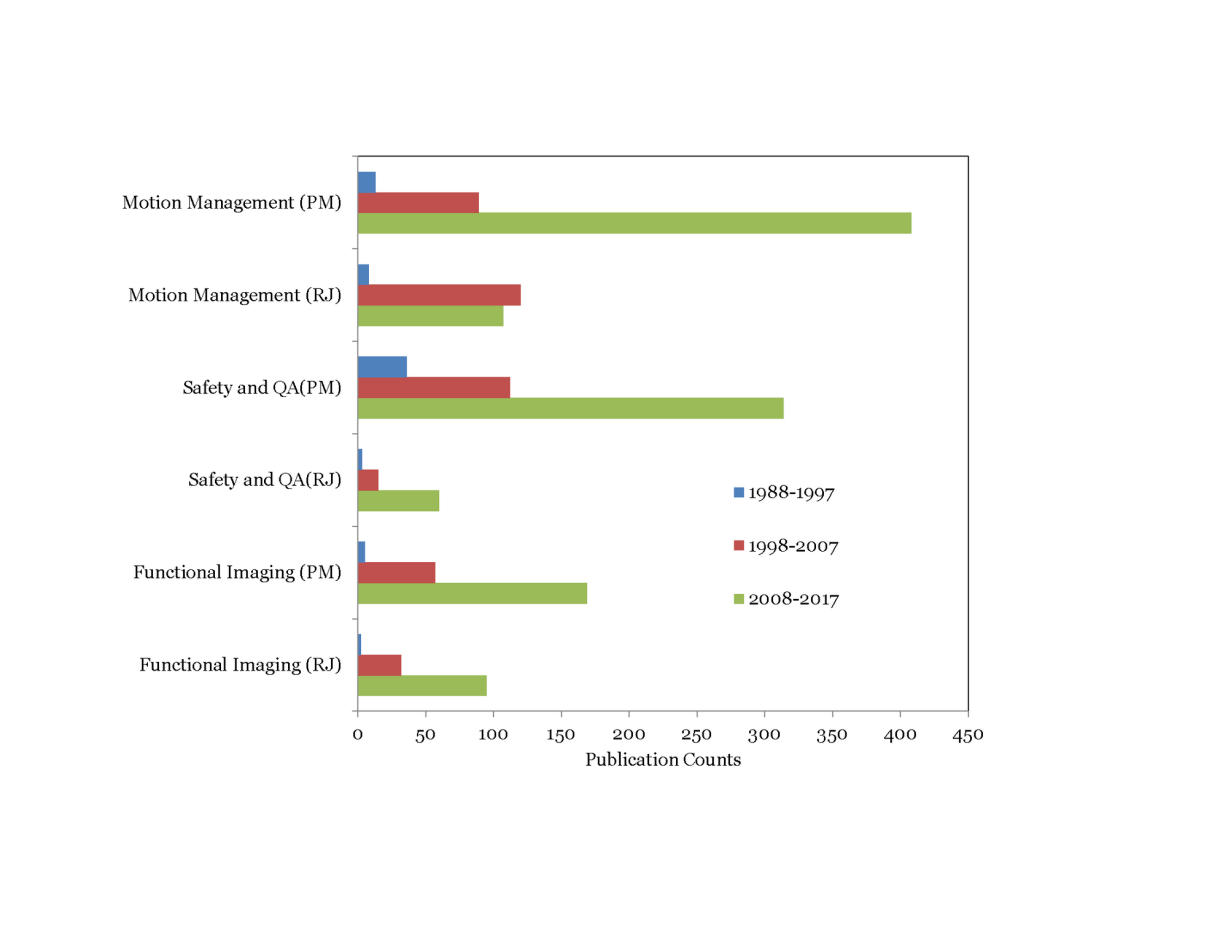
Comparison of physics contributions trends related to other topics of interest between Red Journal (RJ) and PubMed (PM)

Trends in physics contributions observed between the Red journal (RJ) and the PubMed (PM) database on various topics are presented in Figures 5-7.

## Discussion

This review demonstrates that the volume of physics contributions in the Red Journal has increased over the past three decades (Figure 1). The content of these publications appears to mirror timelines in the technological advancement in the field of radiation oncology. The trends are described by as the increase in number of physics contributions on a specific topic of interest immediately following adaptation of the related technology or technique in clinical practice. For instance, preliminary contributions on the topic of 3D-CRT appeared in the years 1987 and 1988 [1-2]; this was followed by 103 publications on the subject of 3D-CRT (simulation + planning+ dose-volume histogram (DVH)) during the years 1988 to 2000. Similarly, IMRT conceptual work appeared in the year 1993 [3], followed by extensive contributions on the applications of IMRT between the years 1999 to the present time (n=204). The references cited herein are examples and not intended to validate the origin of the technology/technique concepts. Spikes in publication trends on other topics were also observed during different times of the study period, which appears to be related again to the technological evolution of the specific application(s) (Figure 4).

What accounts for the trend observed with physics contributions published in the Red Journal? Is it selection bias on the part of the Red Journal favoring physics contributions related to clinical applications of the latest technology or technique at the time of publication? Holliday et al. [4], evaluated characteristics associated with higher rates of acceptance for original manuscripts submitted to the Red Journal. They observed that the acceptance rate for physics/dosimetry/imaging submissions was 31% and cited the h-index of the submitting author was independently associated with higher acceptance rates by the Red Journal. The authors [4], however, did not investigate the study content of submitted physics manuscripts (established vs. state of the art technology and techniques) as one of the characteristics in their study. Holliday et al. [4], based on their study data, concluded that the rejected manuscripts by the Red Journal were eventually published in journals with a lesser impact factor.

Interestingly, we observed that the physics contribution trends in the PubMed database largely mirrored those observed in the Red Journal. Particle beam therapy contributions increased in the third decade while brachytherapy and electron beam therapy contributions decreased (p<0.001) (Figure 5). Contributions on 3D-CRT related topics decreased by 42% in the third decade compared to the second^ ^decade while IMRT related contributions increased three-fold in the third decade compared to the second decade (p<0.001) (Figure 6). As observed in the Red Journal, the proportion of physics contributions focused on outcome/toxicity modeling, motion management, functional imaging for treatment planning, and radiation safety/quality assurance increased in the third decade (Figure 7). One could certainly argue that the publication counts in the PubMed database could certainly be influenced by refining the search terms applied. However, it was our intent to gather information on changes in publication trends on specific topics for comparison purpose and not worry about the accuracy of the publication counts during the time periods on the topics of interest.

With technological evolution of the radiation oncology field to include extensive applications of imaging, application of MRI in radiation therapy (RT) (for planning and delivery), increases in particle therapy applications, and with big-data/machine learning applications and radiomics as currently emerging research areas in radiation oncology [5-8], physics contributions in radiation oncology are undergoing fast transformation from traditional physics contributions such as on treatment planning, dose measurement and optimization studies etc. For instance, roughly 45% of the physics contributions have focused on MRI and particle therapy applications in the issues of the Red Journal published thus far in 2018.

The field of radiation oncology must evolve to fit within the larger oncology landscape. The ASCO (The American Society of Clinical Oncology) named Immunotherapy as the “Advance of the Year” for each of the last two years. It is incumbent upon us to facilitate rigorous exploration of the synergy between radiotherapy and immunotherapy, which has been suggested in case-reports [9] and nonrandomized studies [10]. Additional areas of focus for the future will include particle therapy with heavy ions, novel forms of treatment with unsealed sources, and adaptive treatment planning using new forms of on-board imaging (e.g., MRI).

## Conclusions

In conclusion, trends of physics publications in the Red Journal and globally largely mirror changes in the field of radiation oncology due to technological advances. Our study data reflects a technological transformation of the field over a period of three decades from beam's-eye–view designed static treatment ports to functional imaging and knowledge-based treatment planning with biological dose optimization and real-time tumor tracking capabilities.
